# Effects of Varieties, Producing Areas, Ears, and Ear Positions of Single Maize Kernels on Near-Infrared Spectra for Identification and Traceability

**DOI:** 10.1371/journal.pone.0161489

**Published:** 2016-09-06

**Authors:** Dong An, Yongjin Cui, Xu Liu, Shiqiang Jia, Shuyun Zheng, Xiaoping Che, Zhe Liu, Xiaodong Zhang, Dehai Zhu, Shaoming Li

**Affiliations:** 1 College of Information and Electrical Engineering, China Agricultural University, Beijing, 100083, China; 2 Modern Precision Agriculture System Integration Research Key Laboratory of Ministry of Education, Beijing, 100083, China; 3 Key Laboratory of Agricultural information acquisition technology (Beijing), Ministry of Agriculture, Beijing, 100083, China; 4 Beijing Kings Nower Seed S&T CO., LTD., Beijing, 100080, China; Consiglio Nazionale delle Ricerche, ITALY

## Abstract

The effects of varieties, producing areas, ears, and ear positions of maize on near-infrared (NIR) spectra were investigated to determine the factors causing the differences in NIR fingerprints of maize varieties. A total of 130 inbred lines were grown in two regions in China, and 12,350 kernel samples were analyzed through NIR spectroscopy. Spectral differences among varieties, producing areas, ears, and ear positions were determined and compared on the basis of pretreated spectra. The bands at 1300–1470, 1768–1949, 2010–2064, and 2235–2311 nm were mainly affected by the producing area. Band selection and principal component analysis were applied to improve the influence of variety on NIR spectra by processing the pretreated spectra. The degrees of the influence of varieties, producing areas, ears, and ear positions were calculated, and the percentages of the influence of varieties, producing areas, ears, and ear positions were 45.40%, 42.66%, 8.22%, and 3.72%, respectively. Therefore, genetic differences among maize inbred lines are the main factors accounted for NIR spectral differences. Producing area is a secondary factor. These results could provide a reference for researchers who authenticate varieties, perform geographical origin traceabilities, and conduct maize seed breeding.

## Introduction

Maize is an important source of food and industrial materials. In China, the annual demand for maize seeds is large. However, criminals earn profits by creating fake seeds because the price of seeds with different qualities varies widely; as a consequence, the order of seed market is disrupted, and farmers and seed production enterprises experience significant losses. The quality of a maize seed is mainly related to varieties and producing areas. Seed varieties exhibit different characteristics. Thus, each variety should be identified to prevent counterfeit. Many maize seed-breeding bases are found in Hainan Province, Xinjiang Province, and Gansu Province in China. Different regions are characterized by various environmental factors, such as climate and soil; as such, seeds of the same variety display different characteristics [1]. Producing areas should also be identified to ensure seed quality. Quick, effective, and economic methods have yet to be developed to identify the varieties and origins of maize seeds.

Near-infrared spectroscopy (NIRS) is a rapid, nondestructive, and widely developed low-cost analysis method. A NIR spectrum is a series of electromagnetic waves whose wavelength ranges from 780 nm to 2500 nm; the NIR spectrum contains information regarding hydrogen groups, such as C–H, N–H, and O–H. Therefore, the NIR spectrum can reveal the composition of organic matter in crops; it is also widely used to analyze agricultural products quantitatively and qualitatively [2–5]. In our previous studies, NIRS is applied to identify various commercial maize seeds. The identification models of four varieties have been developed and have yielded an accuracy of >90%; the models can also overcome the influence of seed coat agent and thus can be acceptable for practical applications [6]. Highly accurate variety identification has also been achieved; indeed, genetic factors greatly affect crop composition [7,8], and gene differences among varieties can be detected through NIRS.

Seeds obtained from the same variety but cultivated in different areas differ from one another because of environmental factors, such as climate, temperature, and daylight conditions. Zhao et al. (2013) revealed that the NIR spectra of wheat of the same species cultivated from four areas in China significantly differ; therefore, wheat-producing regions can be identified on the basis of NIRS [9]. In practice, genetic and environmental factors greatly influence the NIR spectra of crop seeds [10,11]. Zhao et al. (2014) analyzed the seeds of 10 wheat varieties harvested from three origins and found that origins and production year, along with genetic factor, greatly affect the NIR spectra of wheat [12].

Differences in genetic materials and growing environments can produce various seed phenotypic traits, which can be detected through NIRS. Therefore, NIRS can be successfully applied to identify the varieties and producing areas of maize. For instance, genetic and environmental factors elicit dual effects on the NIR spectra of maize seeds. However, a definite conclusion on relevant factors has yet to be presented. Likewise, seeds from the same areas can exhibit different traits because of various nutrients among plants and ear positions. However, studies have yet to determine whether the difference between ears and ear positions remarkably influences the NIR spectra of maize seeds.

In this study, the influence degree of genetic factors and environmental factors on NIR spectra of maize seed were firstly studied and compared based large amount samples from different varieties, producing areas. 130 maize inbred lines were harvested from Hainan and Beijing in 2013. For each inbred line, the seeds were collected from five different ears and three different ear positions. The NIR spectra of a seed kernel were obtained and analyzed. The spectral differences of the seeds among inbred lines, producing areas, ears, and ear positions were determined and compared. Thus, this study could determine the main factors that influenced the NIR spectra of maize seeds. This study could also provide a theoretical basis for the identification of the varieties and producing areas of maize seeds on the basis of NIRS.

## Materials and Methods

### Maize Materials and Equipment

A total of 130 maize inbred lines harvested from Hainan and Beijing in 2013 were used in the experiment. No specific permissions were required for plant and harvested maize in Hainan and Beijing. Because these lands are belong to company. We confirm that the field studies did not involve endangered or protected species. Five ears were selected randomly for each inbred line harvested from Hainan. Five seeds were also collected for each position of the ear: ear tip, middle of ear, and bottom of ear. Therefore, 75 (5 × 3 × 5) seeds were collected. For each inbred line harvested in Beijing, 20 seeds were selected randomly. Finally, 9,750 (75 × 130) and 2,600 (20 × 130) seeds were gathered for inbred lines from Hainan and Beijing, respectively.

The NIR spectra of single kernel were obtained with the diffuse reflectance mode of an MPA-type Fourier transform NIR spectrometer (Bruker Co., Germany). The spectrum band scope of the spectrometer ranged from 12000 cm^−1^ to 4000 cm^−1^, with a resolution of 8 cm^−1^ and 1,037 data points. the penetration depth can be 1–5 mm, which can collect abundant information inside seeds. OPUS 6.5 was used to store and transform the spectrum format. The bands at 9000–4000 cm^−1^ (1111–2500 nm) were selected (649 data points) for data analysis because of the large noise of bands at 12000–9000 cm^−1^. The seed embryo was facing the light source and the detector when the spectra of the seeds were obtained. The spectrometer was set to scan 20 times to obtain one spectrum. A Φ22 mm small sample pool was used as an accessory to measure the single kernel. The seeds were cleaned with paper towel and placed in a small sample pool and covered with a gilt lid to prevent light from coming out when a spectrum was obtained. One spectrum was obtained for each seed.

### Spectral Pretreatment

The software employed for the spectra pretreated and principal component analysis was Matlab 8.2 (the USA, Mathworks Company). The spectra were pretreated before analysis was performed because of the complexity, volatility, instability, and weak signal of NIRS. An appropriate pretreatment method is necessary to establish an accurate, reliable, and stable system. In this study, a smoothing treatment was conducted on the original spectra via a sliding average method to eliminate the noise interference of the spectra, with a sliding window width of 9. Subsequently, baseline drift and background interference were eliminated with the first derivative pretreatment, with a difference window of 9, to amplify the spectral difference and to increase resolution and sensitivity [13]. The difference caused by instrument state and sample change was reduced through vector normalization. The treatment method and specific parameters in this part were determined after the experiments were repeated.

### Spectral Feature Extraction

After the original spectra were pretreated, the characteristics of the spectra were extracted through principal component analysis (PCA). The number of principal components (PCs) was determined on the basis of the accumulative contribution rate. The accumulative contribution rate of the first 20 PCs reached more than 98%. Therefore, the first 20 PCs represented over 98% of differences in the data. In this study, the first 20 PCs were selected as the feature data.

### Calculation of the Difference among the NIR Spectra

In this paper, “spectral difference = 1−correlation” was defined as the measure of the influence of a factor on NIRS. A large spectral difference indicated a significant influence. The NIR spectral difference was analyzed on the basis of the following two aspects: spectral difference after pretreatment and spectral difference after characteristic extraction.

A moving window method was adopted to calculate the spectral difference between two spectra. For example, a window was set with a width of odd *n* when the spectral difference between spectra A and B was calculated. The window slid along the spectral data. The correlation of the data vectors of spectra A and B in the window was calculated after each window moved. The spectral difference of the two spectra at the central band point of the window was calculated as 1−correlation. The window sequentially slid backward until the end of the last smoothing window. After optimization was repeated, the determined width of the window was 25.

After the PCA characteristics were extracted, the data of each spectrum were composed of 20 dimensions. The correlation between two 20-dimensional data was calculated. Then, 1−correlation was regarded as the spectral difference between the two spectral data.

### Calculation of the Influence Degrees of Different Factors on NIRS

The NIR spectral difference was considered as the measure to calculate the influence degree of varieties, producing areas, ears, and different ear positions on NIRS. Three other factors remained unchanged when the influence degree of one factor was calculated individually.

The spectra of 130 maize inbred lines from Hainan were selected as the samples to calculate the influence degree of variety. After the spectra were pretreated by smoothing, first derivative, and vector normalization, the difference between the average spectra of each of the two inbred lines was calculated. A total of 8,385 (130 × 129/2) groups of differences were obtained. The average of these differences was regarded as the difference in variety on NIRS, that is, the influence degree of variety on NIR.

For an inbred line, the spectral difference between the average spectra from Beijing and Hainan was calculated after the spectra were treated. The result was considered as the differences in the producing areas of the inbred variety. The average difference of the producing areas of the 130 inbred lines was used as the influence degree of producing areas on NIR.

The data of the samples from Hainan were selected to calculate the influence degree of ears. After the spectra were treated, the spectral differences among five ears of one inbred line were calculated independently. The average of the spectral differences of the ears of 130 inbred line seeds was regarded as the influence degree of ears on NIRS.

The influence degree of different ear positions was calculated using a procedure similar to that of ears.

## Results

### NIR spectral analysis

**Fig 1:**
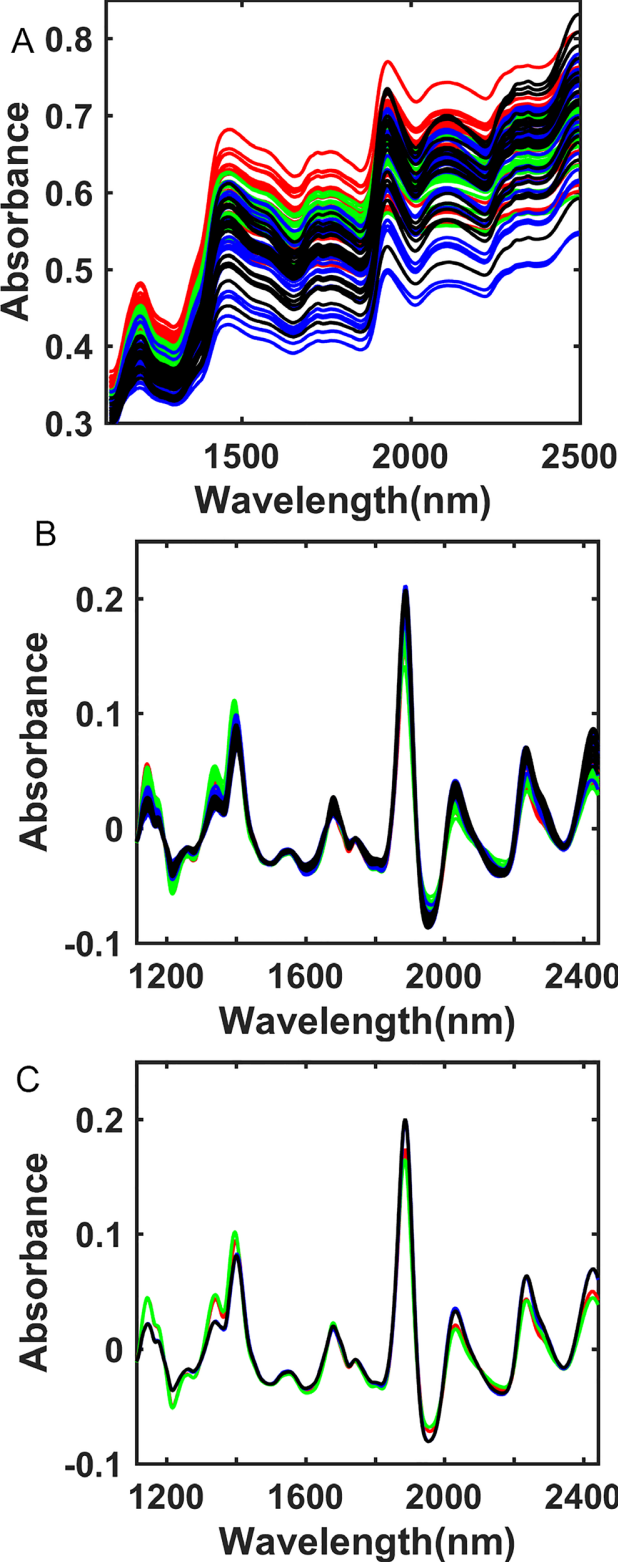
**Source spectra (A), pretreated spectra (B) and average of pretreated spectra (C) of inbred line 1 and 2 harvested from Beijing (BJ) and Hainan (HN).** Red lines represent spectra of inbred line 1 harvested from Beijing. Green lines represent spectra of inbred line 1 harvested from Hainan. Blue lines represent spectra of inbred line 2 harvested from Beijing. Black lines represent spectra of inbred line 1 harvested from Hainan.

The raw spectra data of inbred line 1 and 2 were presented as representation, each inbred line contains seeds which were harvested from Beijing and Hainan. The quality of the spectra were good and we haven't performed any spectra corrections (Fig 1A). Spectra of different kind of samples were overlapped, therefore it is difficult to classify different samples based on raw spectra data. Raw spectra were pretreated by smoothing, first derivative and vector normalization to amplify differences between different kind of samples (Fig 1B).

To observe the result more clearly, the average spectra of these four kinds of samples were calculated and presented (Fig 1C). Spectra of Inbred line 1 and 2 have obvious difference in 1150, 1220,1340, 1390, 1890, 1960, 2030, 2235, 2420 nm, and difference between samples of the same Inbred line harvested from Beijing and Hainan were relatively small. It indicates that influence of gene factors on spectra on spectra are greater than environmental factors between inbred line 1 and 2.

**Fig 2:**
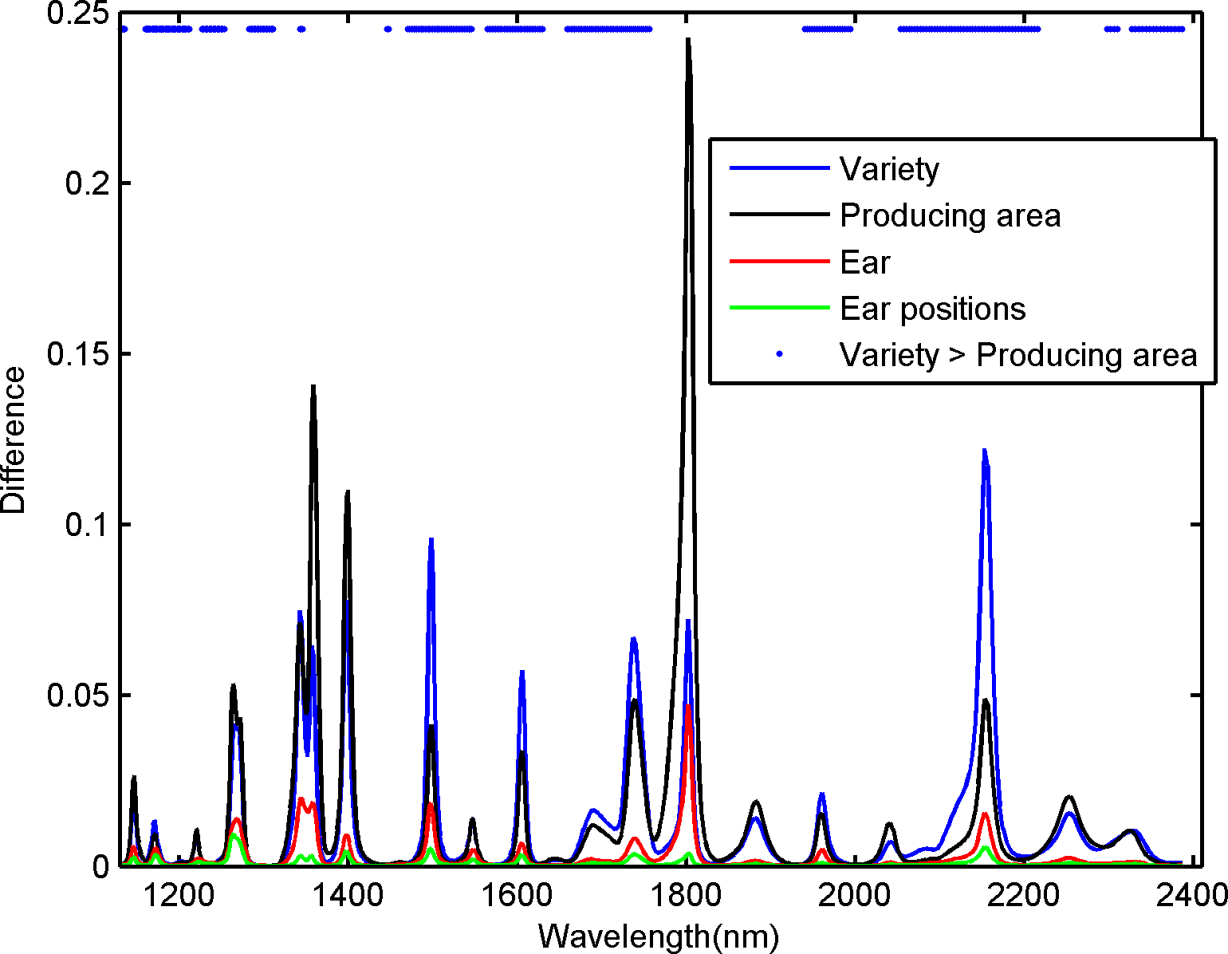
Influence degrees of variety, producing area, ear, and different ear positions at different bands.

The spectral difference was calculated after the spectra were pretreated. The influence degrees of varieties, producing areas, ears, and different ear positions at different bands were then obtained (Fig 2). the band scope ranged from 1130 nm to 2405 nm, with 609 wavelength bands.

The influence degrees of varieties and producing areas were more significant than those of ears and different ear positions in the all-band scope (Fig 2). The influence degrees of varieties and producing areas were basically equal; by contrast, the influence degree of ear was low and the influence degree of different ear positions was the lowest. The influence degrees of each factor significantly varied at different wavelengths. Varieties and producing areas, whose influences were high, were statistically analyzed on the basis of their influence degrees. Table 1 shows the number of wavelength points in different proportionality ranges.

**Table 1:** Ratio between the influence degrees of variety and producing area.

	Ratio	Number of wavelengths
**Variety/Producing area**	3 or more	8
	1–3	309
**Producing area/Variety**	3 or more	35
	1–3	257

Of the 609 bands, 317 (8 + 309) were dominant in terms of variety and 292 (35 + 257) were dominant in terms of producing area (Table 1). The results also revealed 8 bands, whose ratio of the influence of variety to that of producing area was >3. Our data further showed 35 bands, whose ratio of the influence of producing area to that of variety was >3. Moreover, the bands with a dominant producing area (producing area > variety) were mainly distributed at 1300–1470, 1768–1949, 2010–2064, and 2235–2311 nm. In the bands at 1365, 1406, and 1813 nm, the influence of the producing area was more significant than that of the variety.

The sum of the influence degrees of the four factors in different bands was obtained as the measure of their influence on NIRS (for comparison, the spectral difference scope of band 0–8 was mapped to band 0–1). The results are shown in Table 2.

**Table 2:** Influences of the four factors on pretreated NIRS.

Factors	Influence degree	Ratio of influence
**Variety**	0.8111	39.57%
**Producing area**	0.9740	47.52%
**Ear**	0.2010	9.8%
**Ear position**	0.0638	3.11%

For the pretreated spectra, the most significant influence was observed in producing area, followed by variety. The influence of these two factors accounted for 87.09% (39.57% + 47.52%) of the total differences of the NIR spectra, and this influence was higher than that of the two other factors. The influence of ear was 9.8%; by contrast, the influence of the different ear positions was 3.11%.

For the NIR spectra after pretreatment, the influence degrees of the four factors exhibited the following order: producing area > variety > ear > different ear positions. The influence degrees of variety and producing area were 39.57% and 47.52%, respectively. Thus, these factors dominated most of the NIR spectra information.

### PCA

Four inbred lines (No 1, 2, 3, 4) were chosen to show the distribution of samples in the first two PCs. Inbred line 1 and 2 have high separation, inbred line 1 and 3 have typical separation, inbred line 1 and 4 have low separation. In these three cases, different samples of the same inbred line distributed in same area. There are significant difference between samples of different inbred lines.

**Fig 3:**
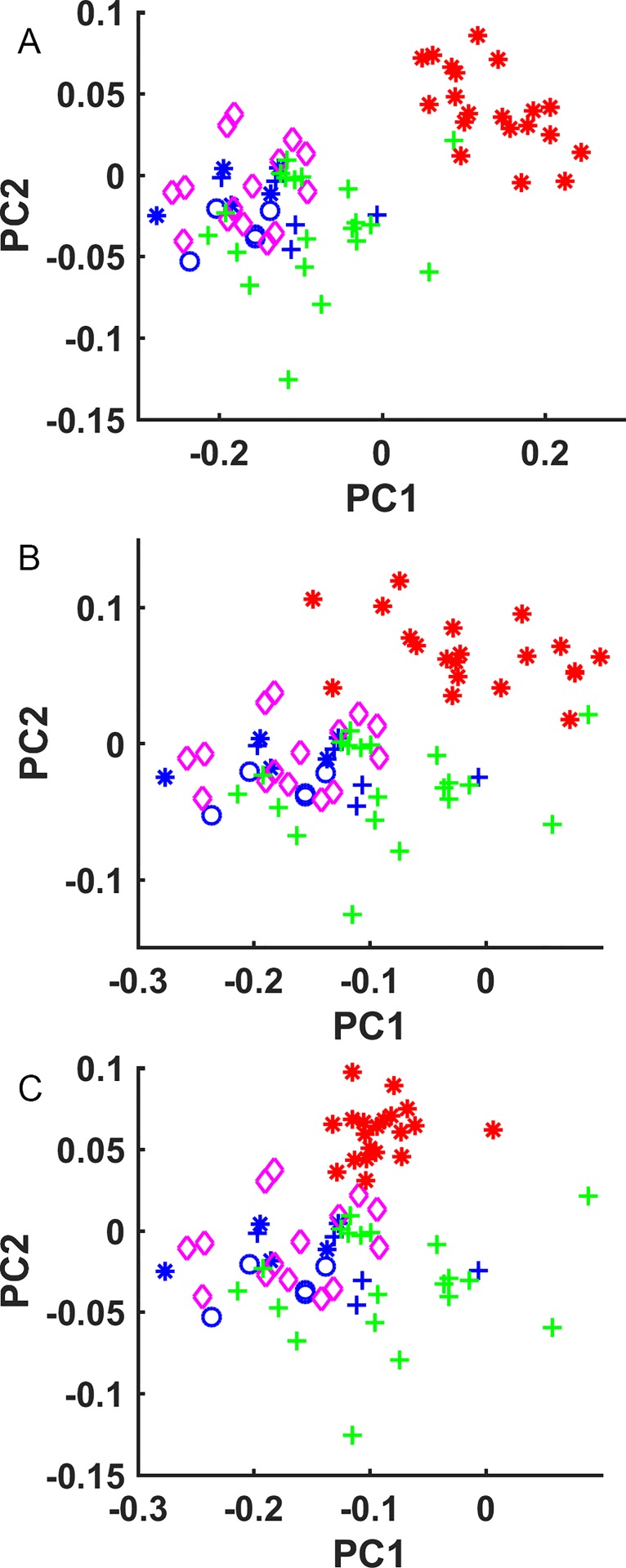
**The first two PCs of the two inbred lines with the high separation (A, inbred line 1 and 2), typical separation (B, inbred line 1 and 3) and low separation (C, inbred line 1 and 4).** Red stars represents inbred line 2, 3, 4 harvested from Hainan in Fig 3A, Fig 3B and Fig 3C, respectively. In Fig 3A, Fig 3B and Fig 3C, blue crosses, stars and circles represent samples collected from top, middle and bottom of ear 1 of inbred line 1 harvested from Hainan. Pink diamonds represent samples collected from ear 2 of inbred line 1 harvested from Hainan. Green crosses represent samples of inbred line 1harvested from Beijing.

The spectrum samples of different maize inbred lines from the same producing areas (Fig 3, blue and pink marks indicated no. 1 maize inbred line of Hainan; red indicated no. 2, 3, 4 maize inbred line of Hainan) were far distributed in the PCA space. They were clearly divided into two parts without creating overlaps among the sample points; this finding revealed a strong distinguishing ability. The spectrum samples of the same maize inbred variety from different producing areas (Fig 3, blue and pink marks indicated no. 1 maize inbred line of Hainan; green indicated no. 1 maize inbred line of Beijing) were divided into two parts in the PCA space, and individual samples overlapped. The distance between the two parts of Beijing and Hainan was shorter than that between nos. 1 and 2 maize inbred lines. This result also showed a distinguishing ability. The spectrum samples of different ears (Fig 3, blue and pink marks indicated ear 1 and ear 2) were overlapping in the distribution area in the PCA space, and the distribution pattern of the spectrum samples of different ear positions (Fig 3, blue crosses, stars and circles) was similar to that of different ears (Fig 3, pink diamonds). This result indicated that different ears and ear positions have little affects on NIR spectra of one inbred line. The pattern observed in other maize inbred lines was similar to that in nos. 1 and 2, 3, 4 maize inbred lines.

After the PCA feature was extracted, the spectral difference was calculated, and the degrees of influence of the four factors on the NIR spectra were considered. Some bands whose difference in varieties was larger than the difference in producing areas were selected and subjected to PCA feature extraction after spectral pretreatment was performed to verify whether the bands with variety dominance (influence of variety > influence of producer) can improve the information proportion of the hereditary factor in the spectrum.

After the features were extracted, the influence degrees of the four factors on the characteristic data exhibited the following order: variety > producing area > ear > different ear positions. The sum of the influence degrees of variety and producing area was 88%. This result indicated that 88% of the characteristic data were related to variety and producing area, and 12% were associated with ear and different ear positions (Table 3). The information proportion was consistent with the distribution of the sample spectra in the PCA space. The influence degree of variety increased by 4.25%, as indicated by the selected bands whose difference in variety was larger than the difference in producing area. By contrast, the influence degree of the producing area decreased by 4.71%. After the bands were selected, the influence degree of variety reached 49.65%, which was nearly half of the total information. This result showed that the bands whose difference in variety was larger than the difference in producing area could be used when NIRS was considered to identify the variety of the maize inbred lines. These bands could also improve the spectral differences among maize varieties, and the influence of producing area on the performance of the model could be reduced.

**Table 3:** Influences of the four factors on PCA data.

Factors	All bands	The bands where variety’s influence is larger
**Variety**	45.40%	49.65%
**Producing area**	42.66%	37.95%
**Ear**	8.22%	8.41%
**Ear position**	3.72%	3.99%

### Influence of producing area on different maize inbred lines

Different maize inbred lines exhibited different genes. The differences in variety and producing areas respectively represented the influence of genes and environmental factors, such as temperature, humidity, and light, on NIRS. Table 3 shows the means of the influence degrees of 130 maize inbred lines. We statistically analyzed 130 maize inbred lines to evaluate the influence degrees of producing area on different maize inbred lines. The spectral differences of bands 0–2 were mapped to bands 0–10 to facilitate the analysis. The results are shown in Fig 3.

**Fig 4:**
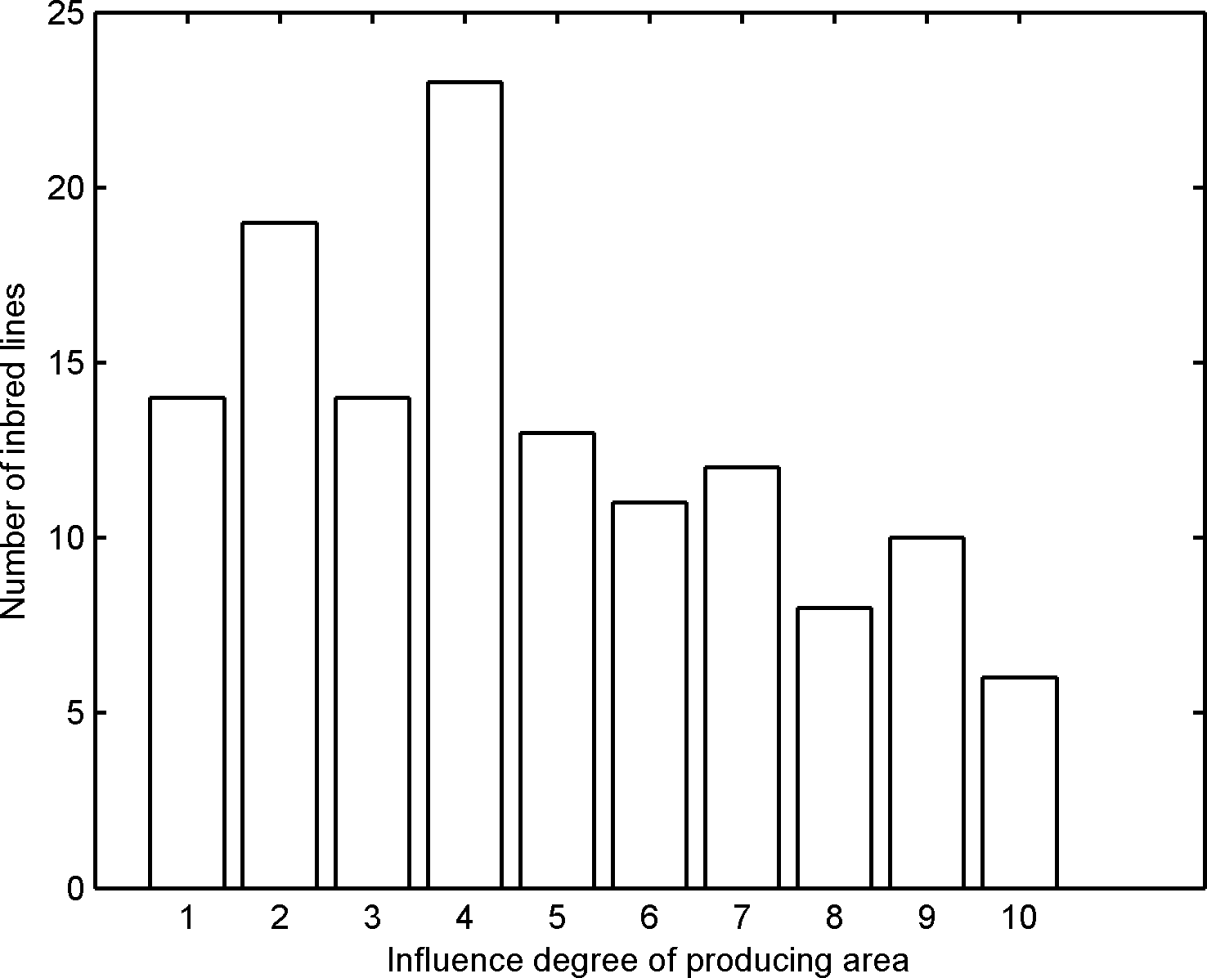
Statistics of 130 maize inbred lines based on the influence degree of producing area.

Most of the NIR spectra of the 130 maize inbred lines were affected by the producing area (Fig 4). The influence degrees of the producing area of 83 maize inbred lines were less than 6. Furthermore, 24 maize inbred lines were significantly affected, and their influence degrees were greater than 7. Therefore, the influence of the producing area differed among various maize inbred lines.

## Discussion

We analyzed the influence of four factors, namely, variety, producing area, ear, and different ear positions, on the NIR spectra of maize inbred lines from the preprocessed spectra and PCA characteristic data by using spectral difference as measure. The results demonstrated that the influences of variety and producing area on the NIR spectra were the most significant, and the sum of their influence degrees was 88%. The sample spectra were distributed in different areas of the PCA space with distinguishing ability. The influence degrees of ear and different ear positions were less than 10%, and their sample spectra were seriously overlapped in the PCA space. Therefore, the NIR spectra of the maize inbred lines mainly revealed the characteristic differences in variety and producing area. The characteristic differences in ear and different ear positions were not evident. Hence, NIRS could be used to identify maize variety and to trace the producing area of maize inbred lines in practice. Producing area could also influence the performance of a variety identification model when variety was identified. The influence of ear and different ear positions could be disregarded when the samples were collected.

Fig 2 shows that the bands with a dominant producing area were mainly distributed at 1300–1470, 1768–1949, 2010–2064, and 2235–2311 nm. Although many bands were dominant in terms of variety, several special bands, such as those at 1365, 1406, and 1813 nm, were considerably affected by producing area. Thus, studies on the producing area traceability of maize inbred lines could be based on the spectral information of these bands, but the interference of these bands should be avoided during the variety identification of maize inbred lines. The spectral difference of varieties was improved to 49.65% after we selected the bands whose difference in variety was greater than the difference in producing area. The difference in producing area was reduced. Therefore, the selection method of bands could be used to increase the spectral difference among varieties and to reduce the influence of producing area when variety was identified.

Compared with the analysis results of the pretreated spectrum, the analysis results after the PCA feature extraction indicated that the influence degree of variety increased by 5.83% (from 39.57% to 45.4%). By contrast, the influence degree of the producing area decreased by 4.86% (from 47.52% to 42.66%). The influence degree of variety was higher than that of producing area. In the pretreated spectra, the influence of producing area was more significant than that of variety. Thus, PCA feature extraction could emphasize the characteristic information of variety, and the influence of producing area on NIR spectra could be reduced.

Furthermore, the influence degree of producing area differed in various maize inbred lines. Several inbred lines were considerably affected by producing area. Conversely, other inbred lines were slightly affected. In seed breeding, maize inbred lines slightly affected by producing area should be selected to reduce the dependence of variety on producing area.

## Conclusions

Genetic differences among maize inbred lines are the main factors accounting for NIR spectral difference, and producing area is a secondary factor. This study can provide a reference for researchers who authenticate maize varieties, investigate geographical origin traceabilities, and conduct maize seed breeding. Environmental factors in different years in a given region vary because of changes in climatic parameters, such as temperature and daylight conditions. Therefore, seeds harvested from different years can exhibit varying traits. The influence of the year of harvest on the NIR spectra of maize seeds will be further investigated.

## Supporting Information

S1 File Near Infrared Sprctra of maize inbred line 1, 2 harvested from Beijing and Hainan. (RAR)
